# Efficacy of Hepatitis B vaccine in those who lost Hepatitis B surface antigen during follow-up: Efficacy of HBV vaccine in those who lost HBsAg

**Published:** 2011-02-01

**Authors:** Hassan Taheri, Mohammad Reza Hasanjani Roushan, Mohammad Jafar Soleimani Amiri, Mohammad Pouralijan, Ali Bijani

**Affiliations:** 1 Infectious Diseases Research Center, Babol University of Medical Sciences, Babol, IR Iran; 2 Razi Laboratory Medical Center, Babol, IR Iran

**Keywords:** Chronic hepatitis B, Follow-up study, HBsAg, Hepatitis B virus, Hepatitis B vaccine

## Abstract

**Background:**

The level of HBsAg in some chronic hepatitis B virus (HBV)-infected individuals may decline over time so that it is not detectable in serum.

**Objective:**

To assess the efficacy of HBV vaccine in those who lost their HBsAg without seroconverssion to anti-HBs antibody.

**Patients and Methods:**

From April 1993 to December 2008, of 1603 chronic HBV-infected individuals, 34 (22 men and 12 women) became HBsAg-negative in follow-up visits, with no detectable anti-HBs antibody and HBV DNA in their sera. They received HBV vaccination at 0, 1 and 6 months (case group). Fifty-two subjects (30 men and 22 women) who were negative for HBsAg, anti-HBs and anti-HBc antibody, received HBV vaccination according to the said schedule (control group). Anti-HBs antibody was assessed one month after the last dose of vaccination in the both groups.

**Results:**

The mean±SD age of the case and control groups was 38±12.7 and 33.4 ± 8.6 years, respectively (p = 0.07). The sex distribution between these two groups were similar (p = 0.652). The mean ± SD years of follow-up for the case group was 7.6 ± 4.5 years. Anti-HBs antibody level ≥ 10 IU/L was found in 8 (24%) subjects in the case group and in 45 (87%) in the control group (p < 0.001). The mean±SD anti-HBs antibody level in the case group was 68 ± 32.66 and in the control group 344.6 ± 38.9 IU/L (p < 0.001).

**Conclusions:**

We found that nearly 24% of chronic HBsAg-positive subjects who lost their HBsAg responded to HBV and the remaining cases need to be followed for occult HBV infection.

## Background

Approximately two billion people in the world have serological evidence of past hepatitis B virus (HBV) infection and more than 400 million people are chronic carriers of HBV. "Chronic carrier" of HBV is defined by the presence of hepatitis B surface antigen (HBsAg) in the serum for more than six months [1][2]. Years after chronic infection, in some cases serum HBsAg level declines below the measurable limit. Although it is unusual, it occurs in less than 1% of adult patients per year and 0.05% to 0.8% of those with infection acquired in infancy or early childhood [2][3]. Detection of HBV DNA in the absence of a detectable HBsAg level and occasionally other HBV serologic markers is termed "occult hepatitis B" (OHB) [4]. These patients not only can transmit HBV to others, but may progress to chronic hepatitis, cirrhosis and hepatocellular carcinoma. The prevalence of OHB in hemodialysis patients in different parts of the world is reported to be 3.8% to 27% [5][6][7][8]. The prevalence and the outcome of OHB in chronic HBV-infected individuals has not yet been reported. On the other hand, those who lost HBsAg and not seroconverted to anti-HBs with no detectable HBV DNA are frequently seen in the clinical practice; the outcome of this group of patients is not clear. This study was therefore performed to evaluate the efficacy of hepatitis B vaccine in subjects who were chronically infected with HBV and lost their HBsAg over time.

## Patients and Methods

### Selection of the case group

From April 1993 to December 2008, 1603 patients with chronic HBV infection were followed at the Department of Internal Medicine and Infectious Diseases, Babol University of Medical Sciences, Babol, Iran. The Departments give service to more than 1.5 million people living in Babol, Amol, and Ghaemshar located in northern Iran. Chronic HBV-infected patients were followed at six-month intervals for determination of their viral status. We provided a follow-up sheet for each person for recording the data during each follow-up. Our follow-up protocol was testing for HBsAg, hepatitis B e antigen (HBeAg) if the patient was HBeAg-positive, antibody against HBsAg (anti-HBs), antibody against HBeAg (anti-HBe), serum aspartate aminotransferase (AST), alanine aminotransferase (ALT), and α-fetoprotein. Liver sonography was performed in those aged over 40 years to find out any evidence of hepatocellular carcinoma. The viral markers were tested by ELISA (HBsAg, from Bio Merieux, the Netherlands; anti-HBs from Radim Italy, anti-HBc, HBeAg from Dia.Pro Diagnostic BioProbes, Italy). These tests were repeated at six-month intervals. Those who lost HBsAg and were not seroconverted to anti-HBs with undetectable serum HBV DNA were retested six months later. For isolation of HBV DNA from plasma, we used QIAamp DNA minikit (QIAGen Gmbh). All processes were done based on the manufacturer's instructions. For quantification of HBV DNA, we used Roter-Geen 3000 (Corbett Research) using Artus HBV RG PCR kit (Qiagen, Humburg, Germany). According to the kit instructions, the sensitivity of the test was 3.8 IU/mL (1 IU = 7 copies/mL). Forty-eight (2.9%) patients became HBsAg-negative. Among them, two developed anti-HBs antibody and 12 had 50 to 850 copies/mL of HBV DNA-both groups were excluded from the study. Therefore, 34 cases (22 men, 12 women) who were HBsAg- and HV DNA-negative but anti-HBe- and anti-HBc-positive in two occasions six months apart, were selected as the case group z (Table 1).

### Selection of the control group

In the control group, 52 healthy individuals (30 men [58%] and 22 [42%] women) were evaluated. Subjects in the control group have never been exposed to HBV (negative for HBsAg, anti-HBs, and anti-HBc).

### Intervention

Both groups received 20 µg hepatitis B vaccine (Engerix-B, SmithKline Beecham) at 0, 1 and 6 months. Sera from all subjects were tested for anti-HBs antibody one month after the last dose of the vaccine. Anti-HBs ≥ 10 IU/L was considered as protective level in both groups. The study was approved by the Infectious Diseases Research Center, Babol University of Medical Sciences. The ethical committee also approved this study. Informed consent was obtained from all participants.

### Statistical analysis

Data were analyzed using SPSS® ver 15. Means of two continuous variables were compared using Student's t test and categorical variables were compared by x(2) test and Fisher's exact test when appropriate. A p-value < 0.05 was considered statistically significant.

## Results

Table 1 shows the outcome of 1603 cases with chronic HBV infection during the follow-up period. Our analysis included 34 cases (22 men and 12 women) who were HBsAg- and HBV DNA-negative but anti-HBe- and anti-HBc-positive. The mean ± SD age of subjects in the case group was 38 ± 12.7 years; it was 33.4 ± 8.55 years in the control group (p = 0.07). The sex distribution in these two groups were similar (p = 0.652). The mean ± SD years of follow-up in the case group was 7.65 ± 4.5 years. ALT and AST in all cases were within the normal range during the follow-up. After vaccination, a protective anti-HBs antibody level (≥ 10 IU/L) was developed in eight (24%) of subjects in the case group and in 45 (87%) in the control group (p = 0001) (Table 2). The non-responders were re-evaluated with no changes in their status. The mean ± SD anti-HBs antibody level in the case group was 68 ± 32.66 and in the control group was 344.6 ± 38.9 IU/L (p < 0.001) (Table 1).

**Table 1:** Selection of the case group

**Characteristics of the cases**	**Group 1 a**No (%)	**Group 2 b**No (%)	**Total **No (%)
	**n = 1523**	**n = 80**	**n = 1603**
**ALT** ≥** 80**(IU/L)	122 (8)	23 (29)	145 (9)
**AST **≥** 80**(IU/L)	76 (4.9)	13 (16)	89 (5.6)
**High levels of α-fetoprotein**	60 (3.9)	2 (3)	62 (3.9)
**Lost HBsAg**	45 (2.9)	3 (4)	48 (2.9)
**Serocoverted to anti-HBs**	2 (0.13)	0 (0)	2 (0.12)
**Detectable HBV DNA**	12 (0.8)	0 (0)	12 (0.7)
**Undetectable HBV DNA**	31 (2)	3 (4)	34 (2.1)

^a^ Group 1: HBsAg- and anti-HBe-positive cases

^b^ Group 2: HBsAg- and HBeAg-positive cases

**Table 2:** Response to hepatitis B vaccine in the two studied groups

**Characteristics of the case and the control group**	**Case group **No.=34	**Control group **No.=52	**p-value**
**Male **No. (%)	22 (65)	30 (58)	0.652
**Female **No. (%)	12 (35)	22 (42)	
**Age **Mean ± SD	38 ± 12.7	33.4 ± 8.5	0.07
**Responded to hepatitis B vaccine **No. (%)	8 (24)	45 (87)	< 0.001
**anti-HBs antibody level **Mean ± SD	68 ± 32.66	344.6 ± 38.99	< 0.001

## Discussion

In this study, the protective anti-HBs level was developed in 24% of individuals who lost HBsAg-a finding not previously reported. Development of anti-HBs antibody may be a clue showing that they have protection and are less likely prone to develop chronic hepatitis, cirrhosis and hepatocellular carcinoma [1][2]. Twenty-six individuals in the case group in our study with no response after hepatitis B vaccination may have low levels of HBsAg, have immunologic tolerance to hepatitis B vaccination, and have no ability to produce anti-HBs antibody as reported previously [9]. In contrast, in the healthy individuals in this study, the response rate was 87%, like what reported earlier [10][11]. The concept of low level HBsAg carriers has further been supported by reports of HBV infection following transfusion of blood from donors with isolated anti-HBc antibody [12]. However, incidental finding of cases with negative HBsAg and anti-HBs antibody and positive anti-HBc antibody are frequently reported in the intermediate and highly endemic regions of HBV infection [11][13][14][15][16][17]. These subjects have either chronic HBV infection but had lost HBsAg over time or resolved HBV infection with decrease in anti-HBs antibody levels below 10 IU/L. With hepatitis B vaccination of those with isolated anti-HBc antibody, several studies reported significant anti-HBs levels in 91%-96% of the subjects [13][14][15][16][17]. In Korea, 34 subjects with persistent isolated anti-HBc antibody who were HBsAg-negative received hepatitis B vaccination at 0, 1 and 2 months; protective level of anti-HBs antibody was developed in 70.6% of them. As we found in our study, the HBV DNA was not detectable in any of subjects with isolated anti-HBc antibody who had not responded to hepatitis B vaccination [10]. Lok, et al, reported no response rate after three doses of hepatitis B vaccine in 28% of 32 subjects with isolated anti-HBc antibody [9]. Lai, et al, reported no anti-HBs response in 22.9% of 48 cases with isolated anti-HBc after three doses of hepatitis B vaccination [18]. Kabir, et al, found that among 94 cases with isolated anti-HBc who received three doses of HBV vaccines, 19 (20.2%) were non-responders. They found that previous history of HBsAg positivity was associated with decreased response to vaccination [19]. The non-responders in the above studies may be due to the presence of low levels of HBsAg in their sera or they might have immunologic tolerance to HBsAg of the vaccine, as we noted earlier. The selection of cases in our study (prior positive HBsAg with loss of this marker over time) compared with those mentioned in the above studies which selected isolated anti-HBc-positive cases may explain the large number of non-responders among our cases. We think that some isolated anti-HBc cases with negative HBV DNA may have OHB. HBV DNA may be present in hepatocytes or other sites and may re-appear later. Investigation of the presence of HBV DNA in hepatocytes of these individuals may be helpful and gives a potential answer to this question. Unfortunately, due to ethical considerations, we did not perform liver biopsy in these cases. At present, an OHB is defined as "isolated anti-HBc cases with detectable HBV DNA measured by PCR" [2]. In this study, we found that nearly 77% of those who lost HBsAg during follow-up and were negative for HBV DNA may still have OHB. The weakness of this study was the relatively small number of patients studied and lack of liver biopsy for detection of HBV DNA in hepatocytes. Another weakness of this study may be related to spontaneous seroconversion in the case group with no relation to vaccination. Other studies with large number of cases with or without vaccination are necessary to confirm our findings. The results of this study emphasize the hypothesis that chronic HBsAg-positive cases who lost their HBsAg and are negative for HBV DNA in their sera may still have OHB.

In summary, the results showed that nearly 24% of chronic HBsAg-positive cases who lost their HBsAg and are negative for HBV DNA responded to hepatitis B vaccination; the remaining people must be followed for OHB.
